# Abundance and distribution of Archaea in the subseafloor sedimentary biosphere

**DOI:** 10.1038/s41396-018-0253-3

**Published:** 2018-08-16

**Authors:** Tatsuhiko Hoshino, Fumio Inagaki

**Affiliations:** 1 Geomicrobiology Group, Kochi Institute for Core Sample Research, Japan Agency for Marine-Earth Science and Technology (JAMSTEC), Nankoku, Kochi 783-8502 Japan; 2 Research and Development Center for Ocean Drilling Science, JAMSTEC, Yokohama, 236-0001 Japan

**Keywords:** Microbial ecology, Environmental microbiology

## Abstract

Subseafloor sedimentary environments harbor a remarkable number of microorganisms that constitute anaerobic and aerobic microbial ecosystems beneath the ocean margins and open-ocean gyres, respectively. Microbial biomass and diversity richness generally decrease with increasing sediment depth and burial time. However, there has been a long-standing debate over the contribution and distribution of Archaea in the subseafloor sedimentary biosphere. Here we show the global quantification of archaeal and bacterial 16S rRNA genes in 221 sediment core samples obtained from diverse oceanographic settings through scientific ocean drilling using microfluidic digital PCR. We estimated that archaeal cells constitute 37.3% of the total microbial cells (40.0% and 12.8% in the ocean margin and open-ocean sites, respectively), corresponding to 1.1 × 10^29^ cells on Earth. In addition, the relative abundance of archaeal 16S rRNA genes generally decreased with the depth of water in the overlying sedimentary habitat, suggesting that Archaea may be more sensitive to nutrient quality and quantity supplied from the overlying ocean.

Marine sediment covers approximately 70% of the Earth’s surface, representing one of the largest microbial habitats on Earth. Previous studies, through scientific ocean drilling, revealed that microorganisms are globally distributed in strictly aerobic sediment columns of the oligotrophic open-ocean gyre [1–4] and in strictly anaerobic organic-rich sediments along the continental margins [5–10], even down to 2–2.5 km below the ocean floor [11–13]. Consequently, the current estimate of the global subseafloor cell number in sediments is 2.9 × 10^29^ cells, corresponding to 4 Pg of biomass carbon (i.e., 0.18–3.6% of the total living biomass on Earth [2]), which is two orders of magnitude lower than the previous estimates [5, 9, 14].

Despite these extensive explorations of microbial biomass in various subseafloor sedimentary habitats over the decades, abundance and distribution of Archaea remain debatable [15]. Considering differences in physiology between Bacteria and Archaea, they could respond differently to environmental settings and thus result in population shift. For example, the more rigid and less permeable cell membrane of Archaea is believed to be favorable for surviving energetically challenging conditions in the deep subseafloor biosphere [16]. Using quantitative real-time PCR with domain-specific primer (and probe) sets, it was reported that the copy numbers of archaeal 16S rRNA genes were several orders of magnitude lower than the total number or even below the quantification limit [7, 8, 17]. However, lack of advanced technology at the time of these investigations could have compromised the accuracy and thus the reliable quantification of archaeal 16S rRNA genes: (1) condensed humic substances were always co-extracted from organic-rich sediment samples, which might inhibit PCR reactions and spectrophotometric quantification [18]; (2) sequence mismatches of the domain-specific primers may not amplify some major archaeal linages [9, 19–21]; (3) DNA extractability between archaeal and bacterial cells may be significantly different [9, 22]; and (4) experimental contaminations easily occur under the standard laboratory condition for molecular microbial ecology [23]. Consequently, compilation of the existing molecular quantification data obtained using multiple methods and different quality controls resulted in highly scattered values [24]. Comparing with the conventional quantitative PCR, digital PCR (dPCR) can relieve the problems (1) listed above because quantity obtained by dPCR is independent from amplification efficiency, and thus that allows direct comparison between different samples from various sedimentary settings.

As an alternative way to assess archaeal abundance in subseafloor sediments, Lipp et al. [9] studied archaeal and bacterial intact polar lipids (IPLs) as live biomarker proxies, showing that at least 87% of IPLs were attributable to archaeal cell membranes, whereas analysis of the relative abundances of archaeal 16S rRNA genes, assessed by slot-blot hybridization and quantitative real-time PCR combined with a physical cell destruction method for DNA extraction, yielded a value of approximately 35–40%. This study pointed out that Archaea contribute to subseafloor sedimentary biomass more than previously expected. However, follow-up radiotracer incubation experiments demonstrated that the degradation rates of archaeal IPLs in sediments are one to two orders of magnitude lower than those of bacterial IPLs, and 50–96% of archaeal IPLs are considered to represent fossil signals [25].

These previous reports suggest that the true nature of archaeal abundance and distribution in the subseafloor sedimentary biosphere remain unclear and should be reconsidered. In the present study, we analyzed 221 sediment cores collected from 0.2–392.2 m below seafloor at 38 drilling sites during 13 scientific expeditions since the first microbiology-dedicated Ocean Drilling Program (ODP) Leg 201 in 2002 (Fig. 1a, Supplementary Table S1). To maintain high levels of quality control and quality assessment for the molecular quantification analysis, we performed sediment sub-sampling, DNA extraction and purification, and quantification of 16S rRNA genes at the same place, at the same time, under consistent experimental conditions (see Supplementary Text).

**Fig. 1:**
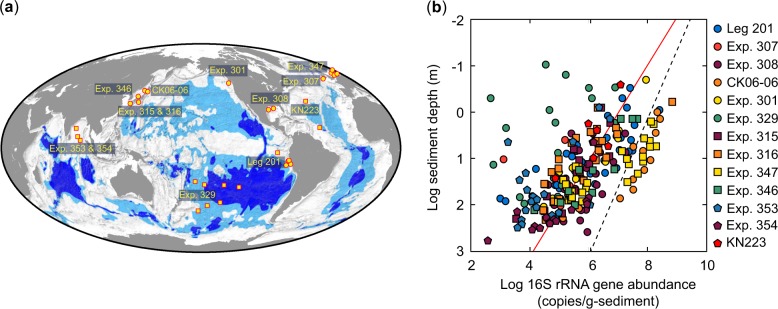
Site locations and 16S rRNA gene abundance. **a** Site locations are plotted on the map showing regions where dissolved oxygen and aerobic activity may occur throughout the sediment [3]. Circles and squares indicate marginal and open-ocean sites, respectively. Sediment samples were collected at different depths from the surface to 392 m below seafloor during 13 scientific drilling cruises from 38 drilling sites. In total, we analyzed 221 sediment samples. Leg 201, Ocean Drilling Program (ODP) Leg 201 Peru Deep Biosphere; Exp. 301, Integrated Ocean Drilling Program (IODP) Expedition 301 Juan de Fuca Hydrogeology; Exp. 307, IODP Expedition 307 Modern Carbonate Mounds: Porcupine Drilling; Exp. 308, IODP Expedition 308 Gulf of Mexico Hydrogeology; Exp. 315 and 316, IODP Expedition 315 and 316 NanTroSEIZE; Exp. 353, IODP Expedition 346 Asian Monsoon; Exp. 347, IODP Expedition 347 Baltic Sea Paleoenvironment; Exp. 353, IODP Expedition 353 Indian Monsoon Rainfall; Exp. 354, IODP Expedition 354 Bengal Fan; CK06-06, the *Chikyu* shakedown cruise offshore Shimokita; KN223, R/V *Knorr* cruise 223 in North Atlantic. **b** Depth distribution of prokaryotic 16S rRNA abundance quantified by microfluidic digital PCR (Supplementary Table S1). The red line indicates the regression line generated using least squares analysis with the abundance of 16S rRNA gene [log (16S rRNA gene abundance) = 7.03 − 0.97 log (depth), *r*^2^ = 0.38]. The dashed black line shows the regression line [log (cell count) = 8.05 − 0.68 log (depth), *r*^2^ = 0.70] of total direct cell count [10]. That regression line is based on cells per mL of sediment instead of copies per gram for dPCR

The abundance of archaeal and bacterial 16S rRNA genes was measured by microfluidic dPCR [18], showing a logarithmically decreasing trend with increasing sediment depth (Fig. 1b). The equation for the regression line relating gene abundance to depth is:

$${\mathrm{Log}} {\mathrm{(16S}}\,{\mathrm{rRNA}}\,{\mathrm{gene}}\,{\mathrm{abundance)}} \\ = 7.03-0.97 \times {\mathrm{log}}\left( {{\mathrm{depth}}} \right){\mathrm{,}}\,r^2 = 0.38$$

which is relatively in good agreement with that of the previous data of acridine orange direct cell count [5, 10]:

$${\mathrm{Log}}\left( {{\mathrm{cell}}\,{\mathrm{count}}} \right) = 8.05-0.68 \times {\mathrm{log}}\left( {{\mathrm{depth}}} \right){\mathrm{,}}\,r^2 = 0.70$$

Those regression lines indicate that the quantity of 16S rRNA gene obtained by dPCR is generally lower than cell counts due to that fact that not full recovery of DNA from sedimentary cells cannot be achieved. By comparing the published data of direct counts of cells and dPCR data in this study, we obtain the following formula between those two data:

$${\mathrm{Log}}\left( {{\mathrm{cell}}\,{\mathrm{count}}} \right) = 1.43-0.92 \times {\mathrm{log}}\left( {{\mathrm{dPCR}}} \right),\,r^2 = 0.41$$

The mean relative abundance of archaeal 16S rRNA genes at each drilling site notably varied (Fig. 2a, Supplementary Fig. S1). The sediment samples used in this study were obtained from 24 marginal ocean sites and 14 open-ocean sites, representing the anaerobic and aerobic microbial ecosystems in the subseafloor sedimentary biosphere, respectively. Our microfluidic dPCR analysis shows that the relative abundance of archaeal 16S rRNA genes to the total 16S rRNA genes (i.e., archaeal and bacterial 16S rRNA genes) is 22.6% and 5.9% in average for the marginal ocean and open-ocean sites, respectively (Fig. 2b). This difference indicates that archaeal contribution to the anaerobic microbial ecosystem is more prominent than to the aerobic ecosystem. The highest archaeal abundance of up to 50.4% was observed at the Integrated Ocean Drilling Program Site 1322 in the Mars-Ursa salt-withdrawal basin on the north-eastern Gulf of Mexico continental slope (Fig. 2a, Supplementary Table S1), where nutrients and energy substrates were additionally supplied from the continent via the Mississippi River to the slope deposit [20, 26].

**Fig. 2:**
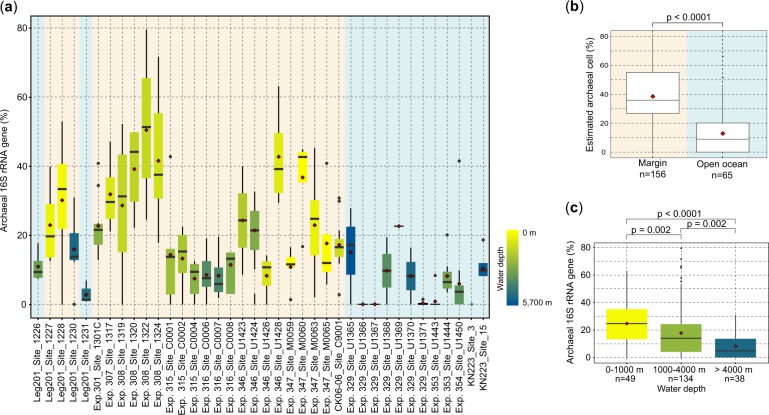
Boxplot of archaeal proportion in microbial 16S rRNA gene (%) at each drilling site determined by microfluidic digital PCR. **a** The edges of the box are the first and third quartile, red diamonds are average proportions, and gray dots are outliers (see also Supplementary Fig. S1, Supplementary Table S1). The color of the box indicates water depth; light blue, marginal ocean sites, and light brown, open-ocean sites. **b** The summary of estimated archaeal cell proportion in marginal ocean sites (*n* = 156) and open-ocean sites (*n* = 65). The relative abundance of archaeal cells to the total microbial cells in marginal ocean sedimentary habitats was significantly greater (Mann–Whitney *U* test, *p* < 0.05) than that in open-ocean sedimentary habitats. **c** The summary of archaeal 16S rRNA gene proportions in the three different water depth classes, 0–1000, 1000–4000, and >4000 m (see also Supplementary Fig. S2). The relative abundance of archaeal 16S rRNA gene in these classes are significantly different (Mann–Whitney *U* test, *p* < 0.05). A high relative abundance of archaeal 16S rRNA genes is observed for the shallow class

To evaluate the population size of archaeal cells based on our dPCR dataset, we applied the average copy number of the 16S rRNA gene on genomes of Archaea and Bacteria, 1.7 and 4.7 copies/genome, respectively (rrnDB version 5.2) [27]. As a result, we estimated that 40.0% and 12.8% of the total microbial cells are Archaea in marginal anaerobic and open-ocean aerobic communities, respectively (Fig. 2b). If we can assume that open-ocean sediments generally contain 10% of all microbial cells in the global subseafloor sediments as suggested by the biogeographic biomass distribution model [2], archaeal cells account for 37.3% of all microbial cells in the global subseafloor sedimentary biosphere. Importantly, despite the variance in archaeal population depending on the oceanographic setting, the relative abundance of Archaea in the subseafloor sedimentary biosphere is relatively similar to the estimate in the global oceans at 41.9% [28]. This suggests that Archaea comprise a biomass comparable to Bacteria throughout the surface and subsurface microbial ecosystem in the ocean.

Upon comparing dPCR data between near-seafloor sediment samples and sediment samples from deeper parts, we identified several sites where the relative abundances of archaeal 16S rRNA genes were higher in deeper horizons than in near-seafloor sediment samples (for example, sites 1226, 1301, 1320, 1322, 1324, M0060, and U1428). This trend was previously observed in IPL-based analyses [9] and other studies of shallow sediments; however, we observed decreasing trends for other sites (Supplementary Fig. S2). Several factors are conceivable for these trends in archaeal fractions: (1) archaeal cells may be more eco-physiologically resistant and/or adaptable to low-energy flux habitats than most bacterial cells [16, 29, 30]; (2) biological and geophysical migration of surface sedimentary conditions may stimulate bacterial growth than archaeal growth [31]; (3) some of Archaea may specifically utilize deeply buried recalcitrant substrates, such as mineralized detrital proteins and/or humic derivatives [32, 33], and therefore, some archaeal fractions may be retained as essential ecosystem functions (for example, methanogenesis and acetogenesis) against the selective environmental pressure of geophysical and energetic constrains during burial [4, 30, 34]; and/or (4) geophysical, sedimentological, and hydrogeological characteristics and formation stability may constrain the supply of water, nutrients, and energy substrates, and subsequently, have an impact on the fraction of archaeal community in subseafloor sedimentary microbial ecosystems [7, 8, 12, 35].

Interestingly, our data also reveal that the relative abundance of archaeal 16S rRNA genes generally decreases with water depth at the drilling site (Fig. 2c, Supplementary Fig. S2). Here we categorized the water depths of study sites into three groups: epi-mesopelagic (0−1000 m); bathy-pelagic (1000−4000 m); and abyss-pelagic depths (>4000 m). Consequently, the relative abundance of archaeal 16S rRNA gene in epi-mesopelagic water depths was found to be the highest at 24.7% among the three depth categories. The relative abundance of archaeal 16S rRNA genes was significantly low (*p* < 0.05, *t*-test) in the deeper bathy- (17.8%) and abyss-pelagic (8.2%) water depths (Fig. 2c, Supplementary Fig. S2). These results suggest that water depth is one of the key environmental factors constraining the relative abundance of Archaea in the total microbial community. One of the possible explanations for why the water depth constrains archaeal population in the subseafloor biosphere may be the quality and quantity of consumable organic matter deposited from the photosynthetic zone in the water column down to the seafloor [36]. Alternatively, the differences in organic matter may cause different consumption rate of oxygen, and therefore  different oxygen concentrations. In addition, any other factor correlated with water depth could also affect archaeal population in the sedimentary habitat. To answer this question, further integrated investigations for evaluating the interaction between organic matter and archaeal metabolic characteristics in the subseafloor sedimentary biosphere are needed [15].

In conclusion, this study provides the best estimates to date of the global distribution and abundance of Archaea in the marginal ocean (anaerobic) and open-ocean (aerobic) sedimentary habitats. Our findings in this global dPCR survey confirm that archaeal biomass significantly contributes to the subseafloor sedimentary biosphere (37.3% of the total microbial cells). Nevertheless, metabolic, physiological, and evolutionary functions of subseafloor sedimentary microbes remain largely unknown. These are the foci of our ongoing research using the global subseafloor sediment core samples.

## Electronic supplementary material

Supplementary Text

Supplementary Figure S1

Supplementary Figure S2

Supplementary Table S1
