# Supplementary material for: Abundance and distribution of Archaea in the subseafloor sedimentary biosphere
Source: ISME J. 2018 Aug 16;13(1):227–31. doi: 10.1038/s41396-018-0253-3 (PMC6298964; doi:10.1038/s41396-018-0253-3)
Supplement: Supplementary file 1 — Supplementary Text [file 41396_2018_253_MOESM1_ESM.pdf]

Supplementary Text

Materials and Methods

DNA extraction

In this study, we used worldwide sediment core samples from the aerobic open-ocean and anaerobic marginal ocean sites (Supplementary Table S1). In total, 221 subseafloor sediment core samples obtained during 13 scientific drilling cruises were analysed in a consistent technical manner. The sediment depth of samples ranged from 0.2 to 392.2 m below seafloor. Deep-frozen sediment core (5 g) was aseptically subsampled from the innermost part of the whole round core without thawing [23, 37]. Subsequently, DNA was extracted by using PowerMax Soil DNA Isolation Kit (Qiagen, Tokyo, Japan) according to the manufacturer's instruction. For ethanol precipitation of DNA, 10 µL of linear polyacrylamide (Thermo Fisher Scientific, Kanagawa, Japan), 12.5 mL of 98% ethanol, and 500 µL of 3 M sodium acetate were added to 5 mL of the eluted DNA solution. After centrifugation at 10,000 ×*g* for 15 min and subsequent washing with 70% ethanol, the obtained precipitate was dissolved in 50 µL of Tris-EDTA solution. For the negative control, 5 mL of MilliQ water, instead of sediment samples, was used.

Quantification of 16S rRNA gene

Quantification of the archaeal and bacterial 16S rRNA gene was performed by microfluidic digital PCR (dPCR) technique as previously described [18] with slight modifications as described below. We used domain-specific primers B27F-B357R [38, 39] and A806F-A958R [40, 41] for bacteria and archaea, respectively. Microfluidic dPCR was performed using BioMark Real-time System and 48.770 Digital Array (Fluidigm, South San Francisco, CA, USA). For each sample, 6 µL of PCR mixture was prepared as follows: 3.0 µL of 2× MightyAmp buffer (TaKaRa Bio, Shiga, Japan), 0.15 µL of Binding Dye sample-loading reagent (Fluidigm), 0.12 µL of MightyAmp DNA polymerase (TaKaRa Bio), 0.3 µL of EvaGreen (Biotum, Fremont, CA, USA), 0.015 µL of Rox dye, 0.15 µL each of forward and reverse primers, 1.0 µL of extracted DNA, and 1.115 µL of water. The PCR was initiated with 2 min at 98˚C followed by 50 cycles of 98˚C for 10 s, 55˚C for 20 s, and 68˚C for 30 s. Subsequently, melting curve analysis was performed to exclude nonspecific amplification from quantification. The lower detection limit of the dPCR quantification was 89 copies of 16S rRNA gene/g-sediment, and all data obtained from 221 sediment core samples were quantifiable without any additional modifications, except for 35 samples in which archaeal 16S rRNA gene was below detection limit. For those 35 samples, we approximated the abundance to be zero for calculating the proportion of archaeal 16S rRNA gene.

References

37. Masui N, Morono Y, Inagaki F. Bio-archive core storage and subsampling procedure for subseafloor molecular biological research. Sci. Drill. 2009;8:35-7.
38. Lane DJ. 16S/23S rRNA sequencing. *In:* Stackebrandt E, Goodfellow M (eds.), Nucleic acid techniques in bacterial systematics. John Wiley & Sons, Chichester, England, pp. 125-175, 1991.
39. Herlemann DP, Labrenz M, Jurgens K, Bertilsson S, Waniek JJ, Andersson AF. Transitions in bacterial communities along the 2000 km salinity gradient of the Baltic Sea. ISME J. 2011;5:1571-9.

40. DeLong EF. Archaea in coastal marine environments. Proc Natl Acad Sci USA. 1992;89:5685-9.

41. Raskin L, Stromley JM, Rittmann BE, Stahl DA. Group-specific 16S rRNA hybridization probes to describe natural communities of methanogens. Appl Environ Microbiol. 1994;60:1232-40.
