# Supplementary material for: Abundance and distribution of Archaea in the subseafloor sedimentary biosphere
Source: ISME J. 2018 Aug 16;13(1):227–31. doi: 10.1038/s41396-018-0253-3 (PMC6298964; doi:10.1038/s41396-018-0253-3)
Supplement: Supplementary file 2 — Supplementary Figure S1 [file 41396_2018_253_MOESM2_ESM.pdf]

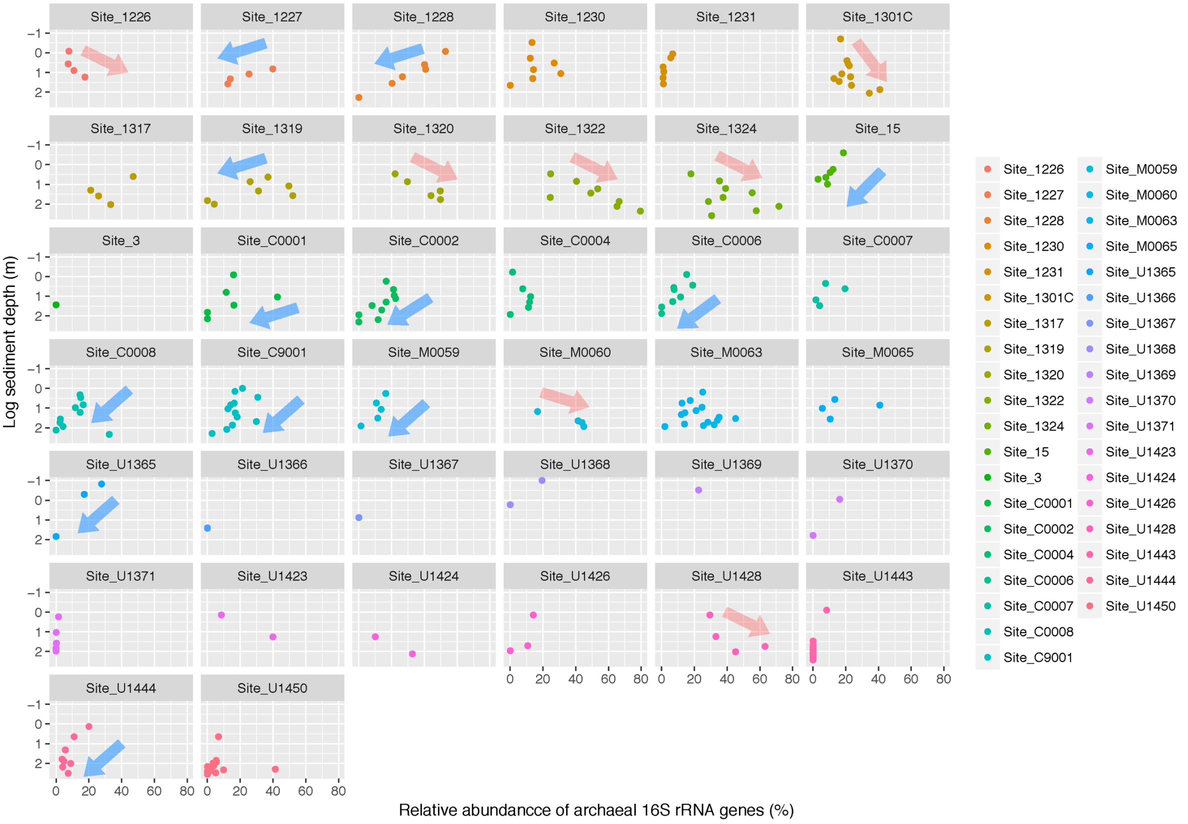

Supplementary Figure S1 Depth distribution of the proportion of archaeal 16S rRNA gene at each sampling site. Red and blue arrows on the plots indicate increasing and decreasing trends of the archaeal proportion with increasing sediment depth.
