# Supplementary material for: Abundance and distribution of Archaea in the subseafloor sedimentary biosphere
Source: ISME J. 2018 Aug 16;13(1):227–31. doi: 10.1038/s41396-018-0253-3 (PMC6298964; doi:10.1038/s41396-018-0253-3)
Supplement: Supplementary file 3 — Supplementary Figure S2 [file 41396_2018_253_MOESM3_ESM.docx]

**Supplementary Figure S2** The relative abundance of archaeal 16S rRNA gene in each sediment sample versus water depth at its sampling site. Circles show samples from marginal ocean sites, whereas triangles indicate samples from open-ocean sites.
